# Correction: Silver nanoparticles from insect wing extract: Biosynthesis and evaluation for antioxidant and antimicrobial potential

**DOI:** 10.1371/journal.pone.0252256

**Published:** 2021-05-20

**Authors:** Parameshwar Jakinala, Nageshwar Lingampally, Bee Hameeda, R. Z. Sayyed, Yahya Khan M., Elsayed Ahmed Elsayed, Hesham El Enshasy

There are errors in the Funding statement. The correct Funding statement is as follows: This study was supported by UGC-RGNF in the form of a fellowship (F1-17.1/2014-15/RGNF-2014-15-SC-TEL-86198) to JP and, DST-PURSE in the form of funding (C-DST-PURSE-II/43/2018) awarded to H B. The authors extend their appreciation for King Saud University, Riyadh, KSA, for funding the work through Researchers Supporting Project (Project No. RSP-2020/52). Kalam Biotech Pvt. Ltd provided support in the form of a salary for YK. The specific roles of these authors are articulated in the ‘author contributions’ section. The funders had no role in study design, data collection, and analysis, decision to publish, or preparation of the manuscript.
