# Supplementary figures and images for: Silver nanoparticles from insect wing extract: Biosynthesis and evaluation for antioxidant and antimicrobial potential
Source: PLoS One. 2021 Mar 18;16(3):e0241729. doi: 10.1371/journal.pone.0241729 (PMC7971846; doi:10.1371/journal.pone.0241729)

**Supplementary material:**

**
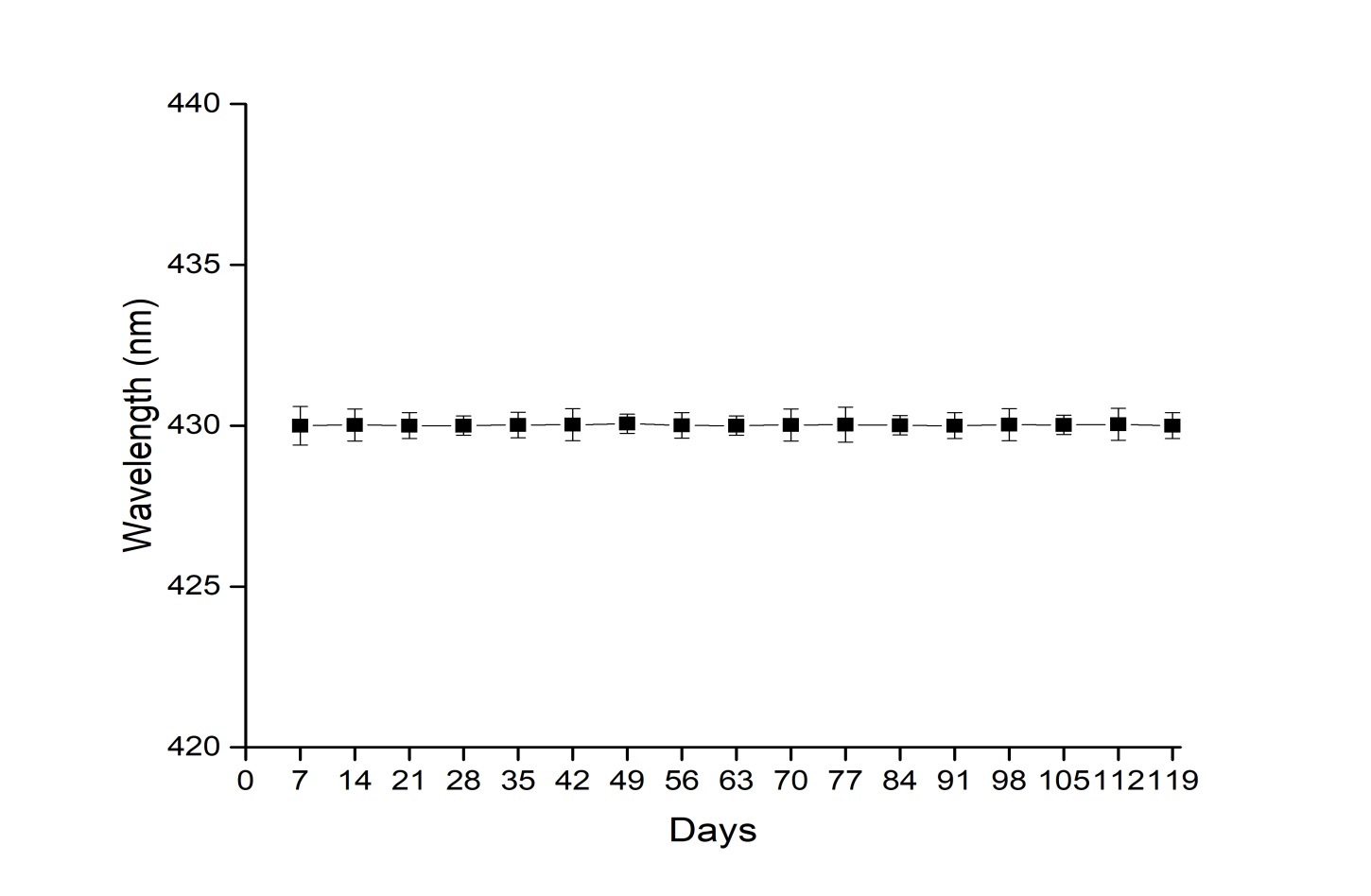
**

**Figure S1** Stability of MMAgNPs

Supplement: S1 Fig — (DOC) [file pone.0241729.s001.doc]

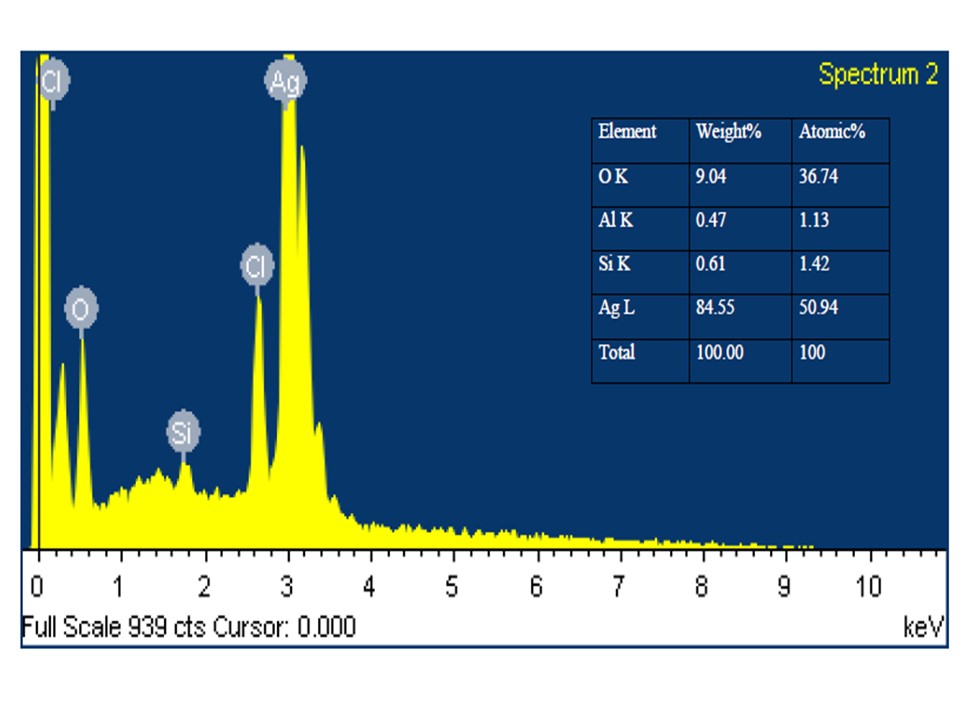
**Figure S2** Energy dispersive X-ray analysis of the synthesized MMAgNPs.

Supplement: S2 Fig — (DOC) [file pone.0241729.s002.doc]

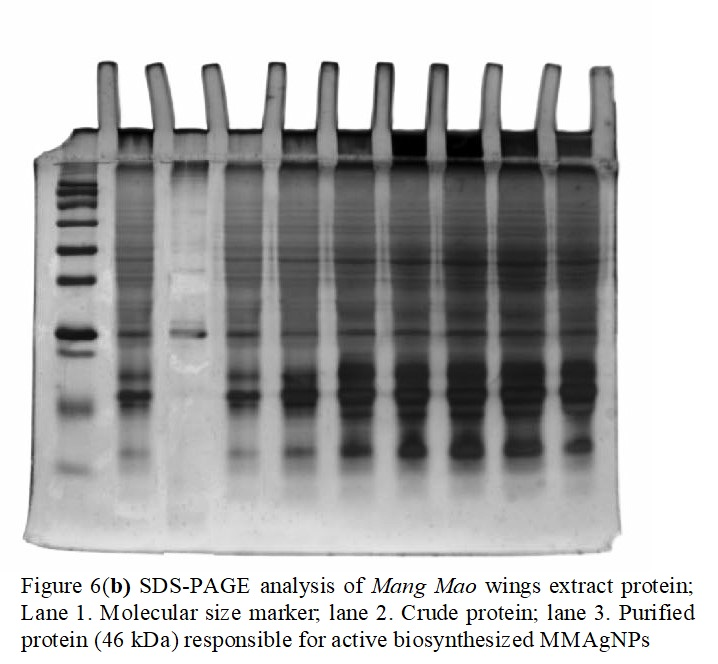

Supplement: S1 Raw Image — (TIF) [file pone.0241729.s007.tif]
