# Supplementary material for: Silver nanoparticles from insect wing extract: Biosynthesis and evaluation for antioxidant and antimicrobial potential
Source: PLoS One. 2021 Mar 18;16(3):e0241729. doi: 10.1371/journal.pone.0241729 (PMC7971846; doi:10.1371/journal.pone.0241729)
Supplement: S3 Fig — (a) Characterization of MMAgNPs by DLS size distribution. (b) Characterization of MMAgNPs by zeta potential analysis. (DOC) [file pone.0241729.s003.doc]

**
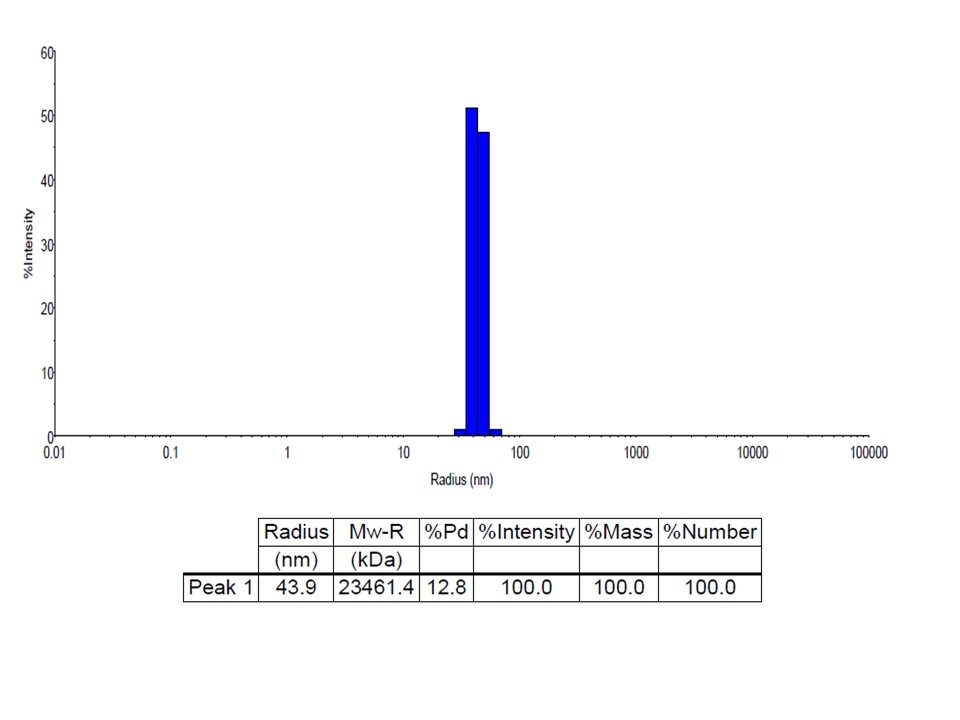
**

**Figure S3** **(a)** Characterization of MMAgNPs by DLS size distribution


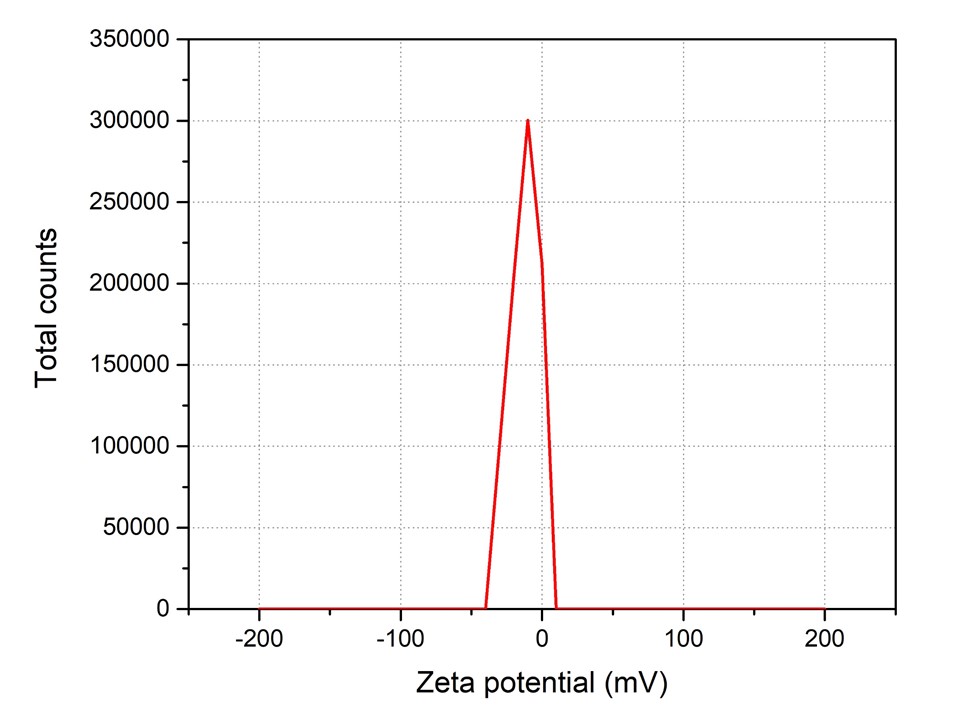


**Figure S3 (b)** Characterization of MMAgNPs by zeta potential analysis
