# Supplementary material for: Silver nanoparticles from insect wing extract: Biosynthesis and evaluation for antioxidant and antimicrobial potential
Source: PLoS One. 2021 Mar 18;16(3):e0241729. doi: 10.1371/journal.pone.0241729 (PMC7971846; doi:10.1371/journal.pone.0241729)
Supplement: S4 Fig — (a) Antibacterial activity of MMAgNPs. (b) Antifungal activity of MMAgNPs. (DOC) [file pone.0241729.s004.doc]

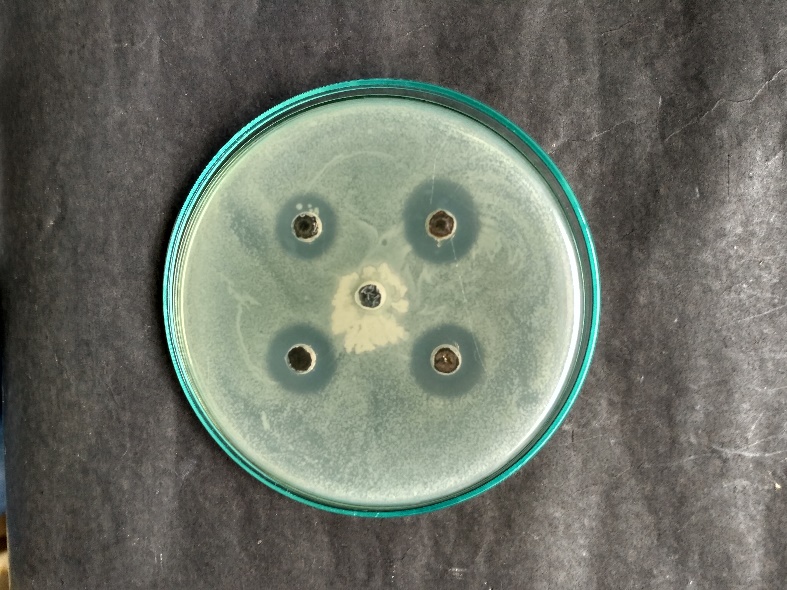


*Escherichia coli* MTCC 43


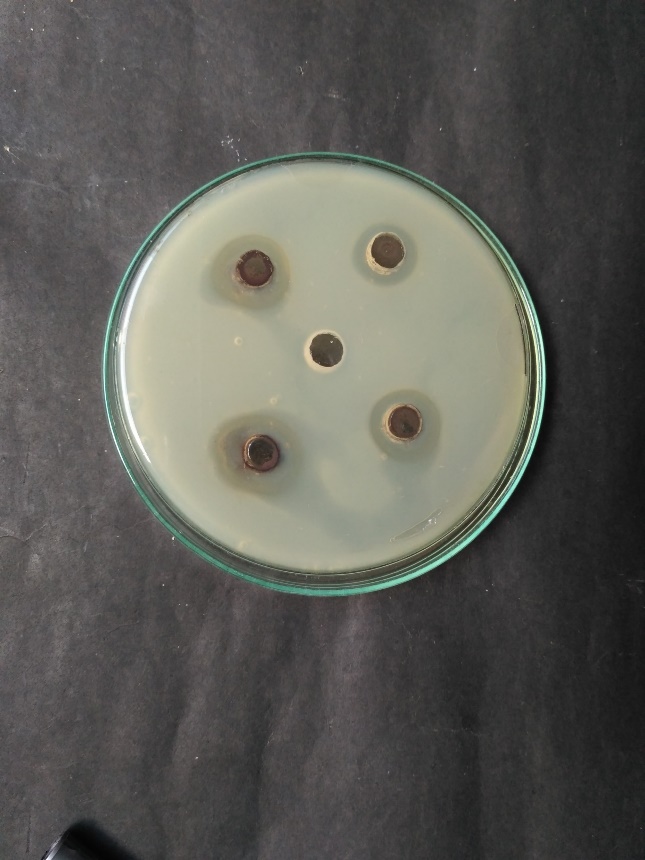


*Achromobacter xylosoxidans* SHB 204

*Pseudomonas aeruginosa* MTCC 424


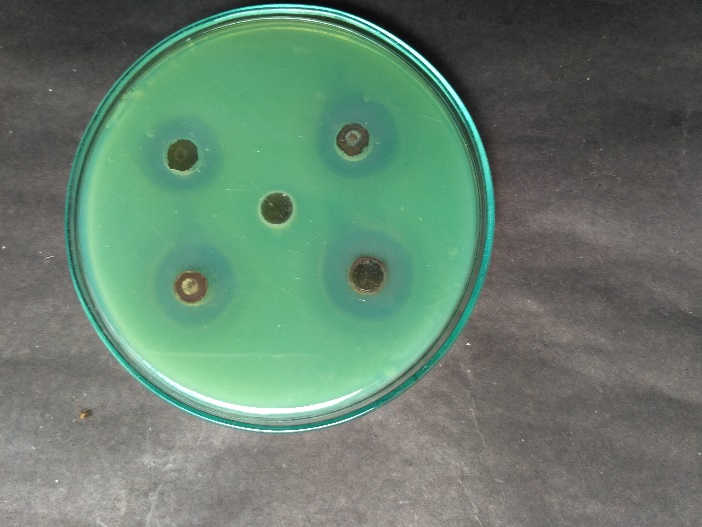

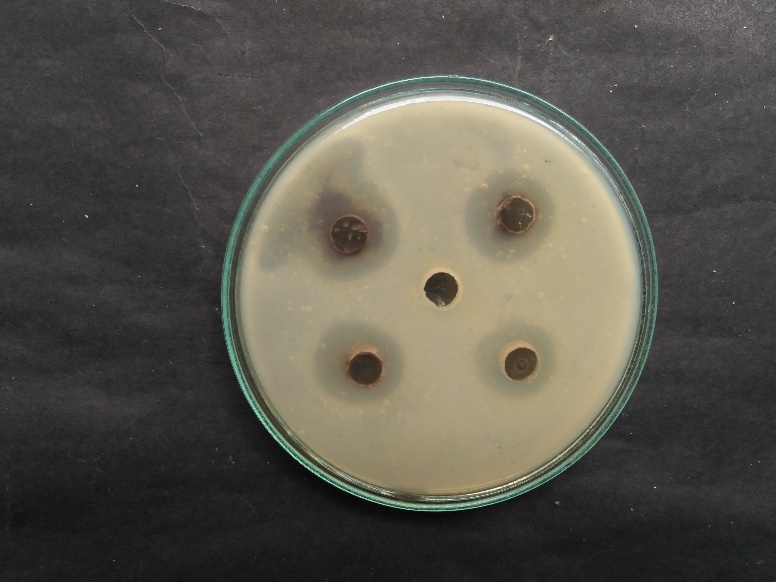


*Klebsiella pneumonia* MTCC 9751


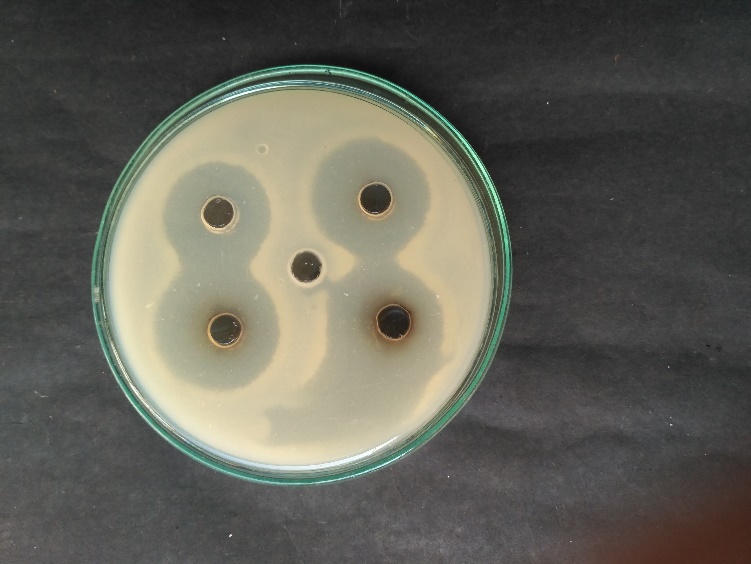


*Staphylococcus aureus* MTCC 96

1

2

1

2

1

2

4

3

4

3

4

3

1

2

1

2

4

3

4

3

**Here : 1= 10µg/ml, 2= 5µg/ml; 3=2.5µg/ml; 4=1.25µg/ml**

**Figure S4 (a)** Antibacterial activity of MMAgNPs


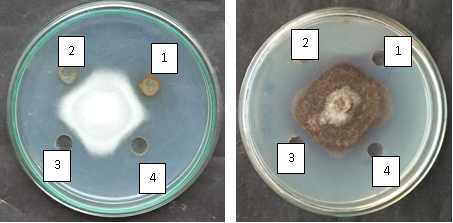

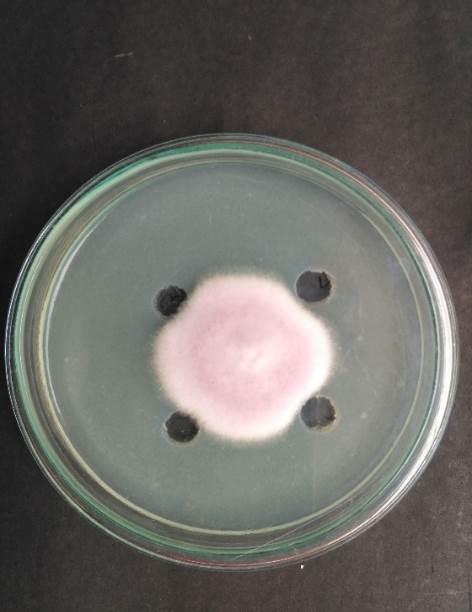

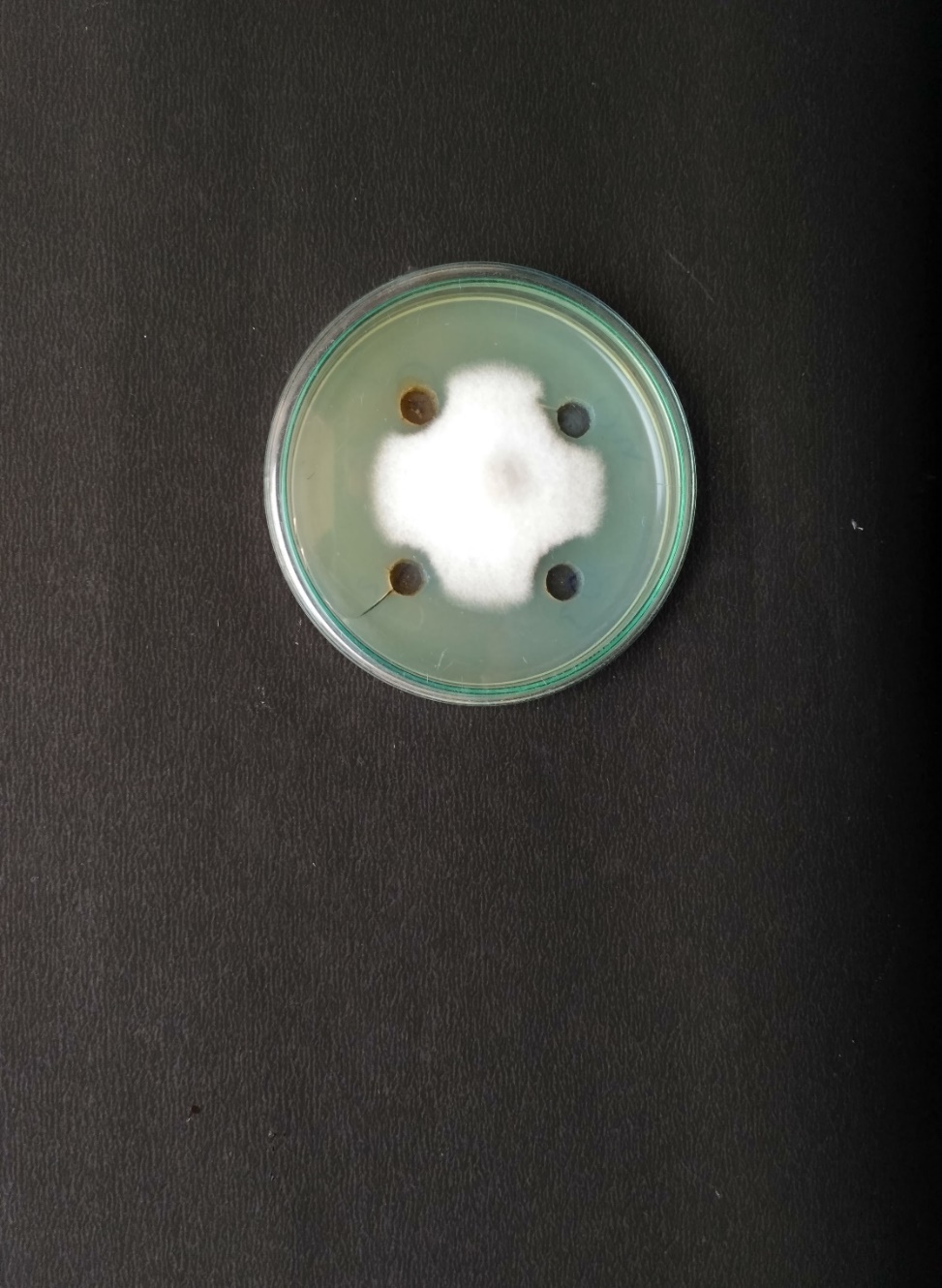


1

4

3

2


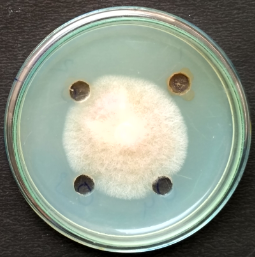


1

2

4

3

***Fusarium oxysporum***

***f.sp. ricini***

***Fusarium oxysporum***

***f.sp. lycopersici***

***Phytophthora nicotianae***

1

2

3

4

***Fusarium sacchari***

***Colletotrichum falcatum***

**Here : 1= 10µg/ml, 2= 5µg/ml; 3=2.5µg/ml; 4=1.25µg/ml**

**Figure S4 (b)** Antifungal activity of MMAgNPs
