# Supplementary material for: Silver nanoparticles from insect wing extract: Biosynthesis and evaluation for antioxidant and antimicrobial potential
Source: PLoS One. 2021 Mar 18;16(3):e0241729. doi: 10.1371/journal.pone.0241729 (PMC7971846; doi:10.1371/journal.pone.0241729)
Supplement: S1 File — (PDF) [file pone.0241729.s005.pdf]

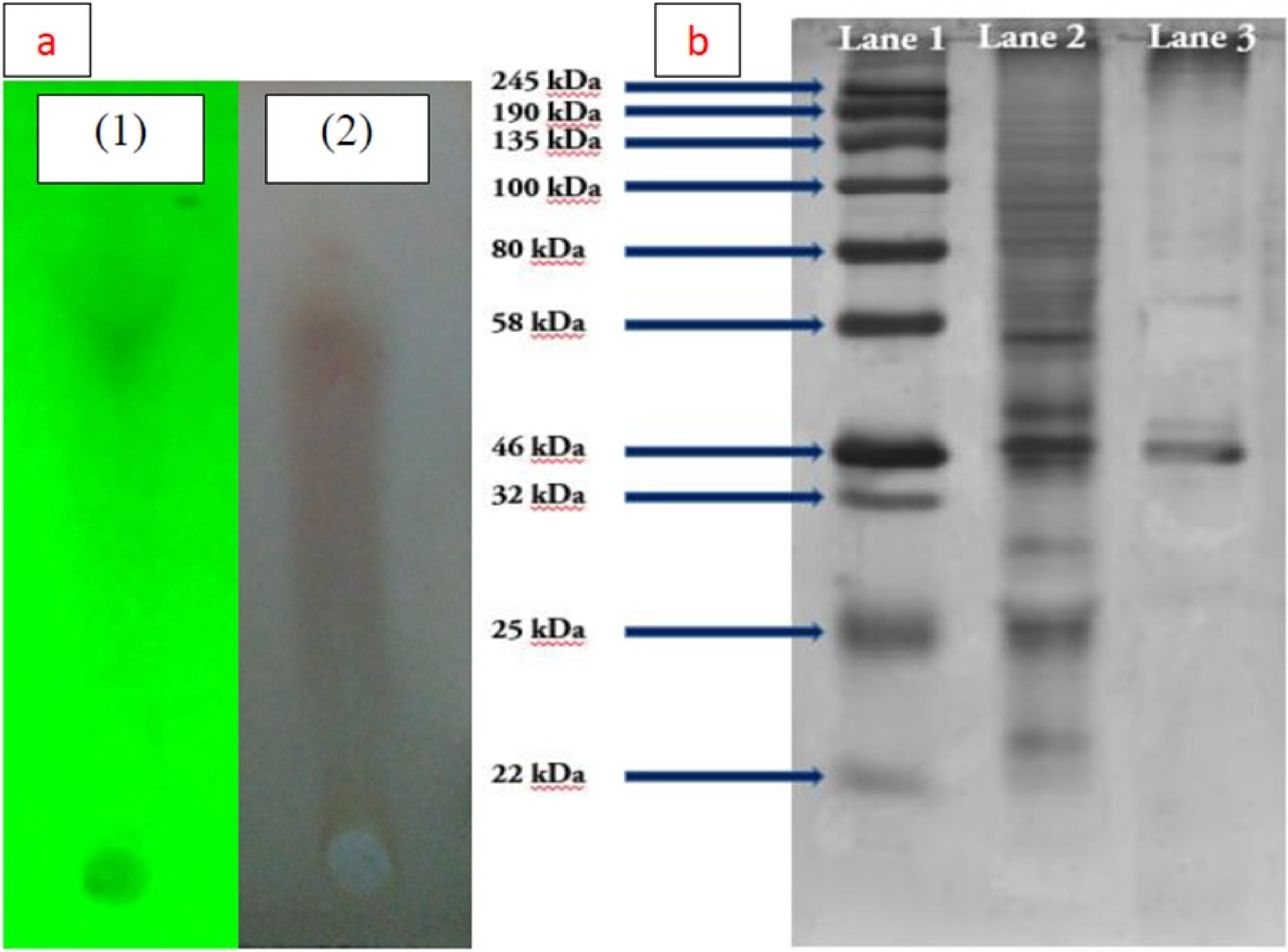
a
(1)
(2)
b
Lane 1
Lane 2
Lane 3
245 kDa
190 kDa
135 kDa
100 kDa
80 kDa
58 kDa
46 kDa
32 kDa
25 kDa
22 kDa

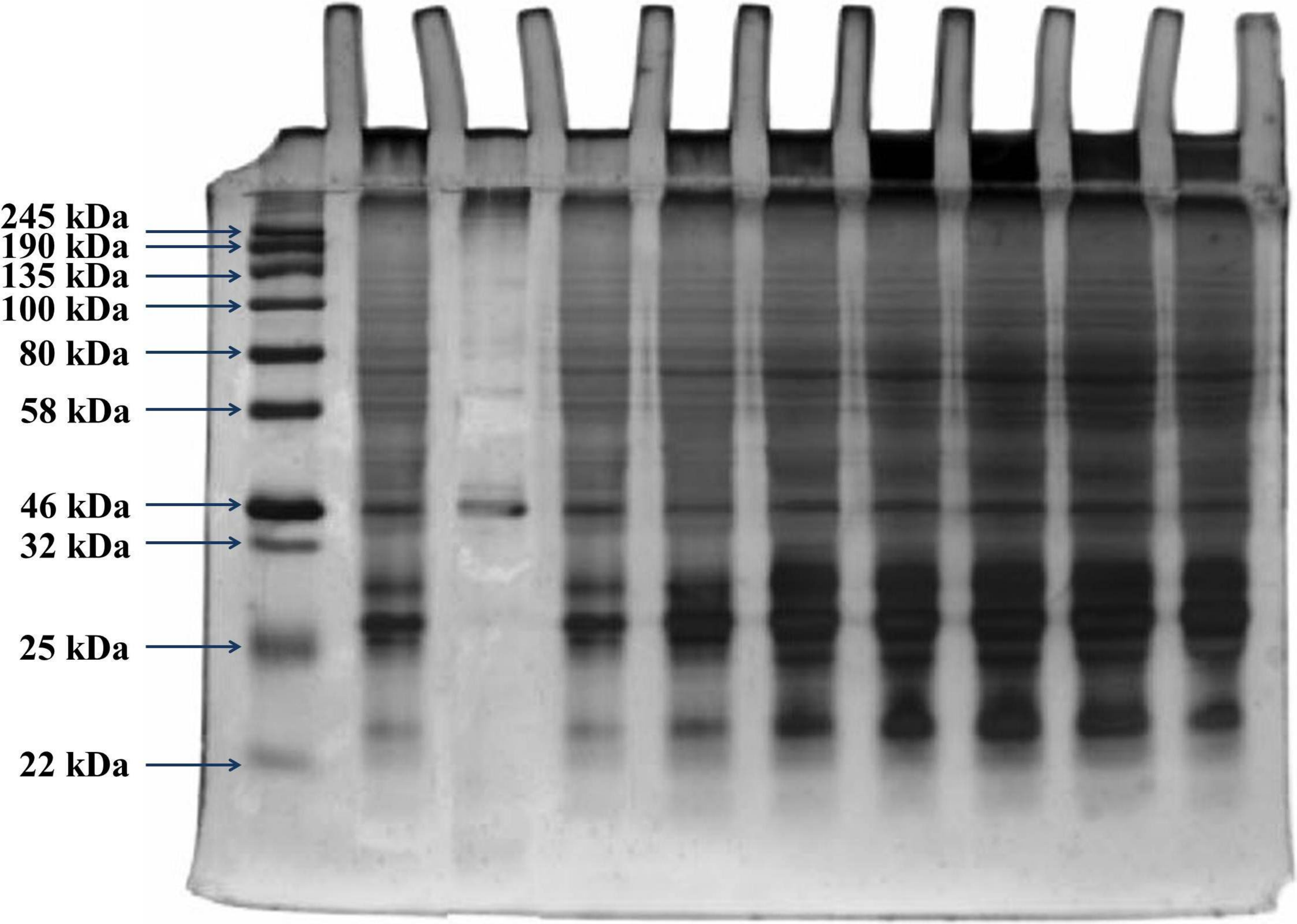

Figure 6(**b**) SDS-PAGE analysis of *Mang Mao* wings extract protein; Lane 1. Molecular size marker; lane 2. Crude protein; lane 3. Purified protein (46 kDa) responsible for active biosynthesized MMAgNPs
