# Supplementary material for: Silver nanoparticles from insect wing extract: Biosynthesis and evaluation for antioxidant and antimicrobial potential
Source: PLoS One. 2021 Mar 18;16(3):e0241729. doi: 10.1371/journal.pone.0241729 (PMC7971846; doi:10.1371/journal.pone.0241729)
Supplement: S2 File — (PDF) [file pone.0241729.s006.pdf]

# Supplementary material:

## Antibacterial activity

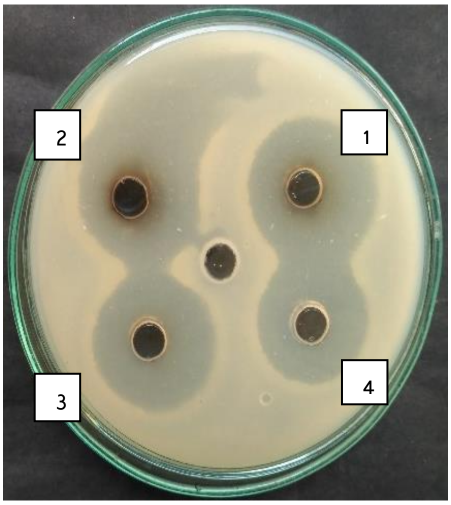


*Staphylococcus aureus* MTCC 96

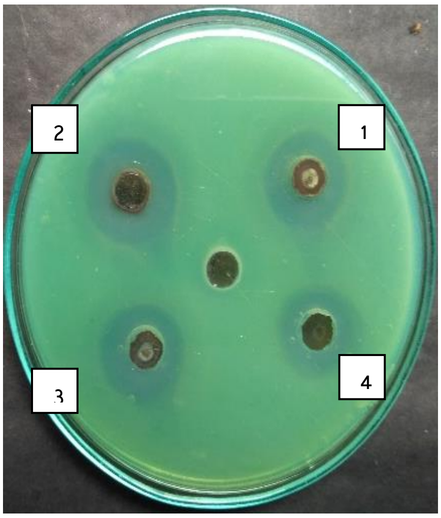


*Pseudomonas aeruginosa* MTCC 424

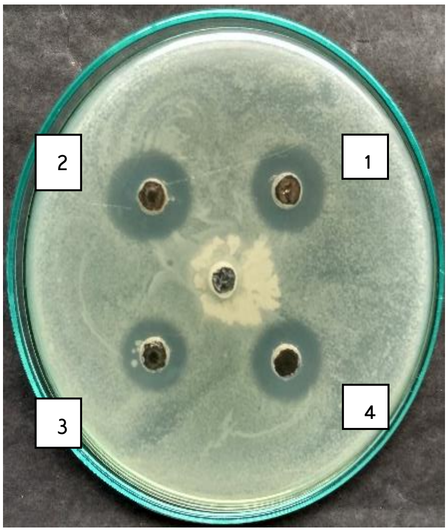


*Escherichia coli* MTCC 43

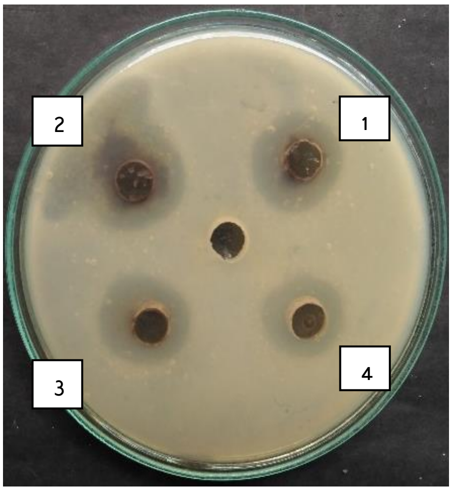


*Klebsiella pneumonia* MTCC 9751

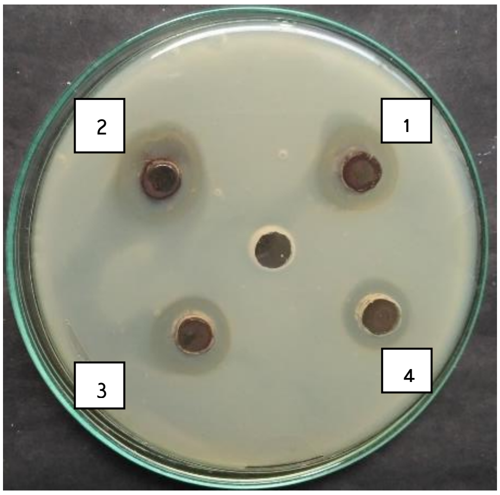


*Achromobacter xylosoxidans* SHB 204

**Here : 1= 10µg/ml, 2= 5µg/ml; 3=2.5µg/ml; 4=1.25µg/ml**

**Antifungal activity**

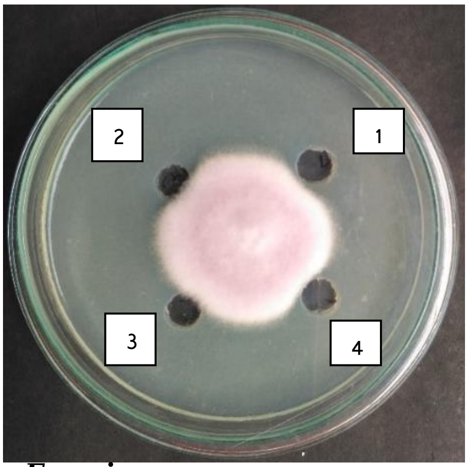


*Fusarium oxysporum f.sp. ricini*

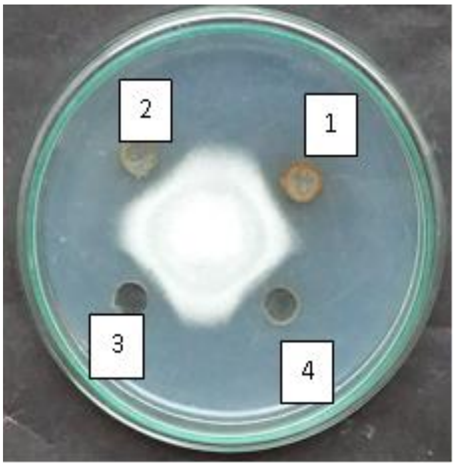


*Fusarium oxysporum f.sp. lycopersici*

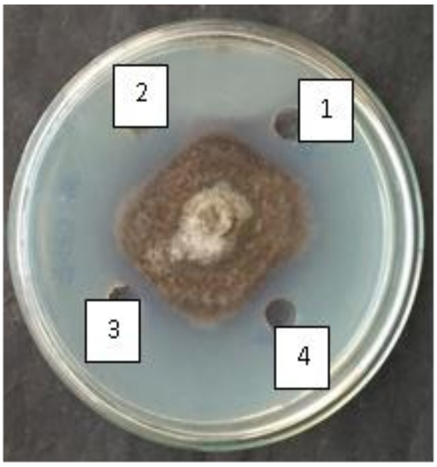


*Phytophthora nicotianae*

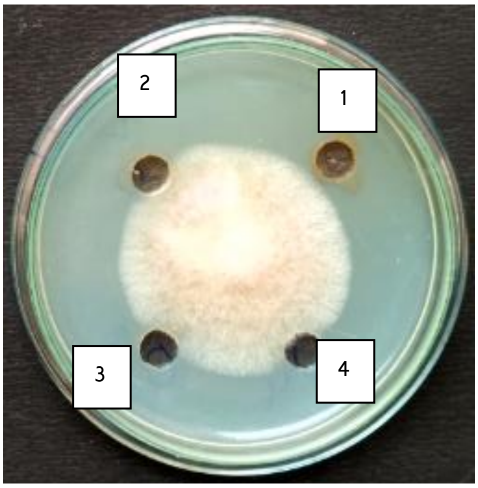


*Fusarium sacchari*

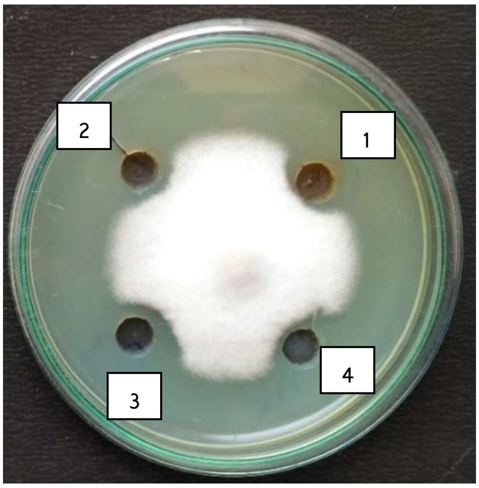


*Colletotrichum falcatum*

**Here : 1= 10µg/ml, 2= 5µg/ml; 3=2.5µg/ml; 4=1.25µg/ml**

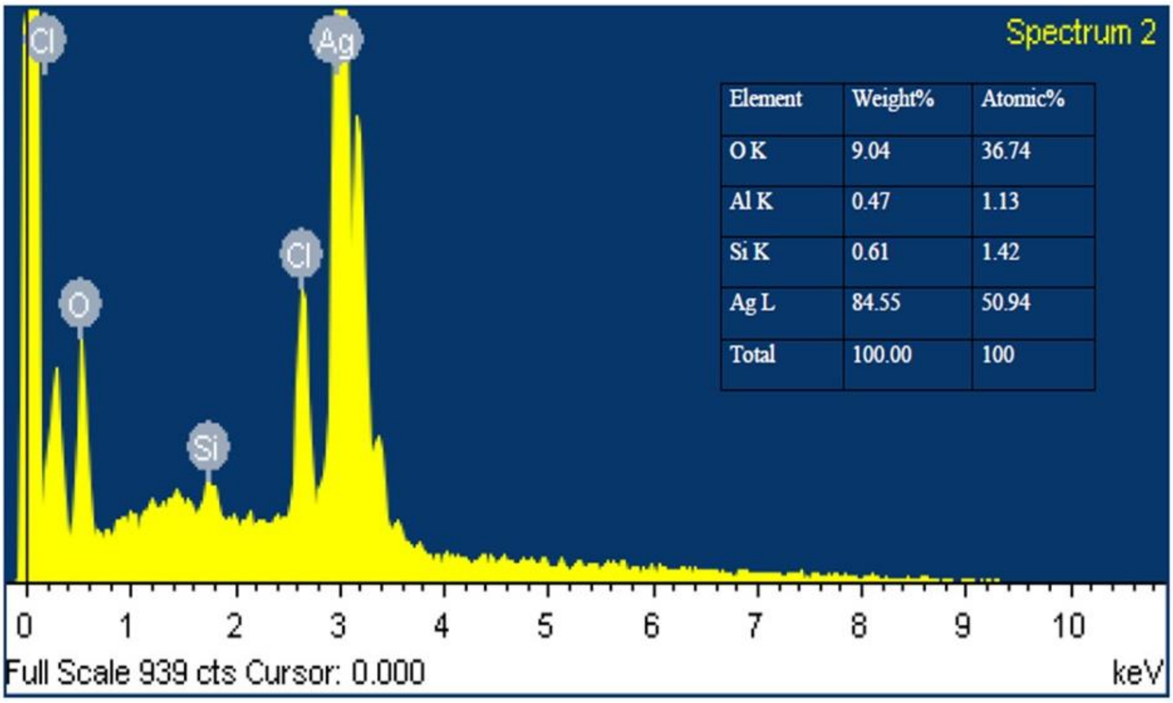


**Figure 4 (a)** Energy dispersive X-ray analysis of the synthesized MMAgNPs.

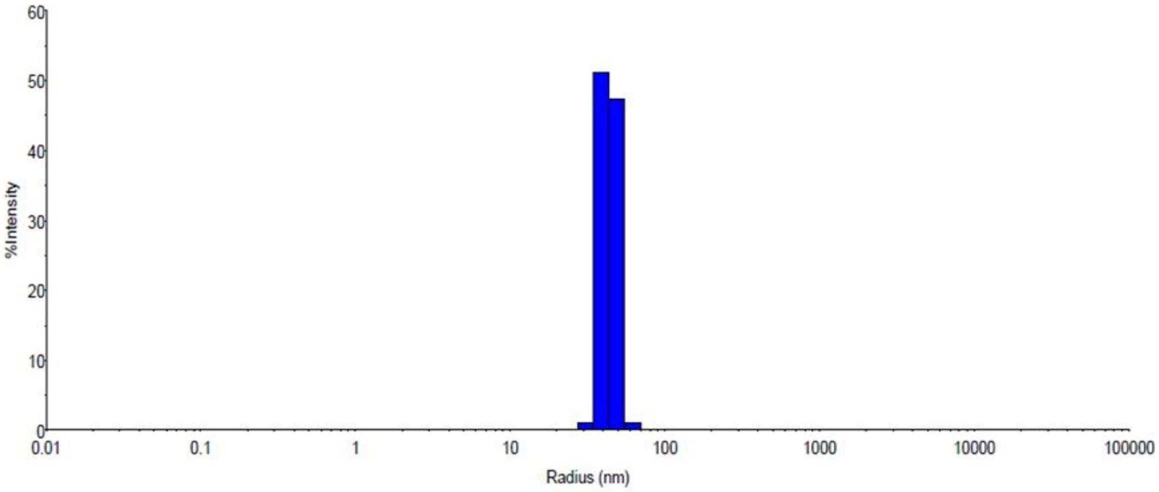


| | Radius (nm) | Mw-R (kDa) | %Pd | %Intensity | %Mass | %Number |
|---|---|---|---|---|---|---|
| Peak 1 | 43.9 | 23461.4 | 12.8 | 100.0 | 100.0 | 100.0 |

**Figure 5** Characterization of MMAgNPs by DLS size distribution

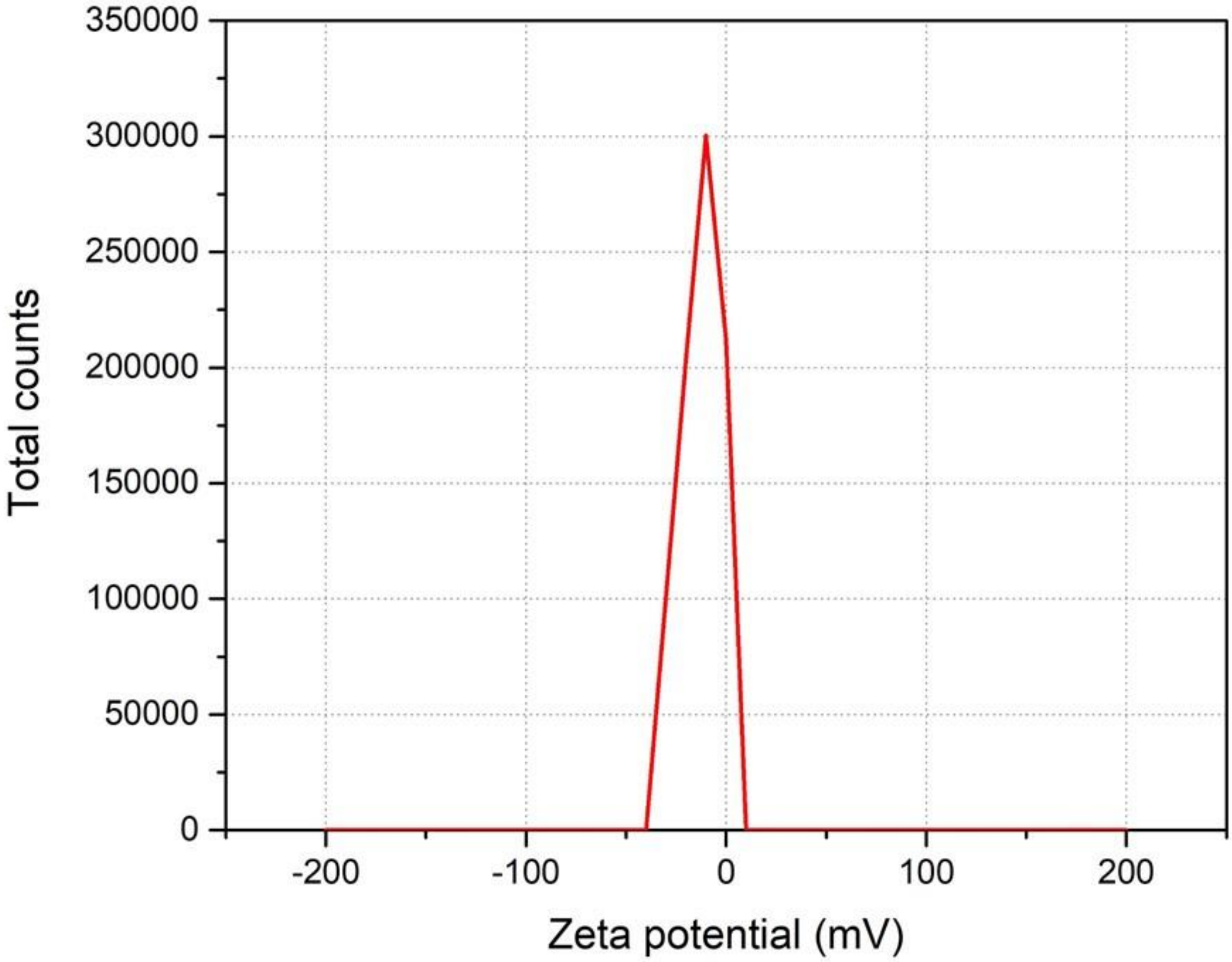


**Figure 5** Characterization of MMAgNPs by zeta potential analysis
